# The Relationship between the Therapeutic Alliance and Suicidal Experiences in People with Psychosis Receiving Therapy

**DOI:** 10.3390/ijerph182010706

**Published:** 2021-10-12

**Authors:** Charlotte Huggett, Patricia Gooding, Gillian Haddock, Daniel Pratt

**Affiliations:** 1 Division of Psychology and Mental Health, School of Health Sciences, University of Manchester, Manchester Academic Health Science Centre, Zochonis Building, Brunswick Street, Manchester M13 9PL, UK; Patricia.A.Gooding@manchester.ac.uk (P.G.); gillian.haddock@manchester.ac.uk (G.H.); daniel.pratt@manchester.ac.uk (D.P.); 2 Greater Manchester Mental Health NHS Foundation Trust, Manchester Academic Health Science Centre, 3rd Floor, Rawnsley Building, Manchester Royal Infirmary, Hathersage Road, Manchester M13 9WL, UK

**Keywords:** therapeutic alliance, suicide, psychological therapy, psychosis, dose of therapy

## Abstract

Few studies have examined the relationship between the therapeutic alliance in therapy and suicidal experiences. No studies have examined this relationship with people with non-affective psychosis. The present study sought to redress this gap in the literature. Sixty-four participants with non-affective psychosis and suicidal experiences who were receiving a suicide-focused cognitive therapy were recruited. Self-reported suicidal ideation, suicide plans, suicide attempts, depression, and hopelessness were collected from participants prior to starting therapy. Suicidal experience measures were collected again post-therapy at 6 months. Therapeutic alliance ratings were completed by clients and therapists at session 4 of therapy. Dose of therapy was documented in number of minutes of therapy. Data were analyzed using correlation coefficients, independent samples *t*-tests, a multiple hierarchical regression, and a moderated linear regression. There was no significant relationship found between suicidal ideation prior to therapy and the therapeutic alliance at session 4, rated by both client and therapist. However, there was a significant negative relationship between the client-rated therapeutic alliance at session 4 and suicidal ideation at 6 months, after controlling for pre-therapy suicidal ideation, depression, and hopelessness. Furthermore, the negative relationship between the client-rated alliance and suicidal ideation was the strongest when number of minutes of therapy was 15 h or below. A stronger therapeutic alliance developed in the first few sessions of therapy is important in ameliorating suicidal thoughts in people with psychosis. Nevertheless, it is not necessarily the case that more hours in therapy equates to a cumulative decrease in suicidal ideation of which therapists could be mindful. A limitation of the current study was that the alliance was analyzed only at session 4 of therapy, which future studies could seek to redress.

## 1. Introduction

Suicide is a major global health concern and is the leading cause of death in people with non-affective psychosis [1,2]. Suicidal experiences including thoughts, urges, plans, attempts and death are known to be amplified in those with non-affective psychosis [2,3,4]. In this population, factors such as male sex, ages 26–45, current suicidal ideation, previous suicide attempts and depression have been found to be significant risk factors for death by suicide [5,6,7,8,9]. Additionally, depression and hopelessness are well-known predictors of suicidal ideation and suicide attempts [3,5,7,10,11,12,13]. More severe experiences of psychosis have also been associated with more severe suicidal ideation and behaviors [3,10,14]. Both suicidal ideation and plans are known to be associated with immense psychological pain, which can lead to suicide attempts and greater risk of suicide death [15,16].

A meta-analytic review indicated that cognitive-based therapies were effective in reducing suicidal thoughts, plans and attempts compared to treatment as usual (TAU), including those with non-affective psychosis [17]. Building on this work and grounded in contemporary transdiagnostic psychological models of pathways to suicidal experiences [18,19,20], Cognitive Behavioral Suicide Prevention therapy for people with psychosis (CBSPp) [16,21] addresses both suicidal experiences and non-affective psychosis. Two pilot randomized controlled trials demonstrated that CBSPp was feasible to deliver and acceptable for people with non-affective psychosis at risk of suicide [21,22]. Additionally, CBSPp was effective in improving suicidal ideation and hopelessness related to suicide compared to TAU [21]. Therefore, there is a growing evidence base for psychotherapies which are tailored specifically for the treatment of suicidal ideation and/or behavior in the context of non-affective psychosis.

When examining the effects of psychological therapy in people with non-affective psychosis and suicidal experiences, two crucial therapy process factors to consider are the therapeutic alliance and ‘dose’ of therapy. The therapeutic alliance is defined as the development of a client-therapist bond and collaborative agreement on goals and tasks for therapy [23]. Client experiences prior to therapy may influence the development of the client-therapist alliance [24]. Psychosis and suicidal experiences are often associated with high levels of self-stigma, which could act as a barrier to disclosure and discussions in therapy [16,25,26,27,28]. The literature so far remains inconclusive as to whether the client’s experiences of psychosis [29,30] or suicidal thoughts/behavior prior to receiving therapy impact upon the therapeutic alliance that subsequently develops during therapy [31,32].

A wealth of evidence has indicated that a therapeutic alliance established early on in psychological therapy is related to positive outcomes for people with a range of mental health problems [33]. For people with psychosis, four studies included in a recent literature review showed that a stronger therapeutic alliance, perceived by clients and therapists, was related to improvements or less severe symptoms of psychosis, whereas five studies have failed to find evidence for such a relationship [29]. Nevertheless, a more robust therapeutic alliance, viewed by the client, was related to increased feelings of hope for the future, desire to seek help and quality of life [29]. For people with suicidal experiences, a stronger therapeutic alliance achieved during therapy was consistently related to a greater reduction in suicidal thoughts post-therapy [31,34] and suicide attempts over the course of therapy [35]. Therefore, the therapeutic alliance during psychological therapy appears be an essential facilitator of improved outcomes for people with either suicidal experiences or psychosis.

The effect of dose of therapy on therapeutic outcomes is important. However, there is a dearth of literature on the optimum dose for therapy for mental health problems despite some evidence suggesting that a greater number of sessions is linked with more positive client outcomes [36,37]. It is likely this is variable; however, such information may be crucial for planning and delivery of services. Dose of therapy comprises several components, such as, number, duration, quality and/or content of therapy sessions [37] and has been linked to the strength of the therapeutic alliance. More specifically, in CBT for psychosis (CBTp), a stronger, client perceived, therapeutic alliance, and a higher number of sessions attended were related to improved symptoms of psychosis [36]. However, if clients perceived the therapeutic alliance as poorer, and also attended a higher number of sessions, this was related to increased symptoms of psychosis [36]. Such findings suggest that assessing the strength of the therapeutic alliance when examining the optimum dose of therapy is critical to preventing potential harm in psychological therapy. Relatedly, another fundamental factor is whether the content or quality of therapy sessions and the therapeutic alliance are related to poorer, or even harmful, outcomes. Therapists and researchers should routinely monitor and assess whether adverse effects and/or unintended harm in therapy has occurred [38].

It is encouraging that some studies have focused on the pertinent issue of the therapeutic alliance in relation to either symptoms of psychosis or suicidal experiences, i.e., ideation, plans, and attempts. That said, there is a major omission in the literature of studies examining the relationship between the therapeutic alliance in therapy and suicidal experiences (pre- and post-therapy) and the potential role of ‘dose’ of therapy, in people with non-affective psychosis [39]. The present study was a secondary analysis of data collected from participants with non-affective psychosis who were randomly allocated to the intervention arm of the Cognitive AppRoaches to coMbatting Suicidality (CARMS) two-armed RCT, which compared TAU plus CBSPp therapy to TAU alone [40].

The aim of the current study was to investigate the relationship between the therapeutic alliance in CBSPp and suicide ideation, plans and attempts in people with non-affective psychosis. Three research questions were posed, 1. Is there a relationship between suicidal experiences prior to starting therapy and the therapeutic alliance? 2. Does the therapeutic alliance predict suicidal experiences measured post-therapy? 3. Does the number of minutes of CBSPp therapy session attendance amplify the relationship between the therapeutic alliance and suicidal experiences post-therapy?

In relation to the research questions, it was hypothesized that, (1) client suicidal experiences (ideation, plans and attempts) prior to therapy will be related to the therapeutic alliance (client and therapist rated); (2) the therapeutic alliance (client and therapist rated) will be negatively associated with suicidal experiences (ideation, plans and attempts) post-therapy (part A), whilst controlling for suicidal ideation, depression, and hopelessness at baseline (part B). (3) The negative relationship between the therapeutic alliance and suicidal experiences post-therapy will be amplified by dose of therapy (total number of minutes of therapy sessions).

## 2. Materials and Methods

### 2.1. Participants

At the time of this study, 101 participants were randomly allocated to the intervention arm of the CARMS trial [40]. The CARMS trial was registered with ClinicalTrials.gov (identifier NCT03114917) and the ISRCTN registry (reference ISRCTN17776666). Ninety-one participants gave consent to take part in the secondary analysis study, of which a complete data set was available for 64 participants. Reasons for missing data (*n* = 27) include, clients did not attend 4 sessions of therapy (*n* = 10), client and therapist WAIs were not completed (*n* = 13) and post-therapy ASIQ was not completed (*n* = 4). In order to assess whether the group of client’s whose data were excluded/missing were similar or different to the group whose data were included, bootstrapped independent samples *t*-tests (for continuous outcome data) and chi squared tests (for categorical outcome data) were conducted. There were no significant differences in age (*t*(89) = 0.89, *p* = 0.357, 95% CI (−2.79, 8.56)), gender (*X*^2^(1, *N* = 90) = 0.106, *p* = 0.745), baseline suicidal ideation (*t*(89) = 0.81, *p* = 0.448, 95% CI (−9.57, 23.64)), baseline suicide plans (*X*²(1, *N* = 86) = 0.906, *p* = 0.341) and baseline suicide attempts (*X*^2^(1, *N* = 90) = 1.33, *p* = 0.249), between clients whose data was included and excluded/missing from the study. Therefore, the final sample comprised 64 participants who provided outcome data prior to the COVID-19 pandemic and subsequent lockdown regulations [41]. Participants’ mean age was 36.83 years (SD = 13.92, range = 19.61–65.62). Just over half of the participants identified as male (*n* = 35), with one participant preferring not to disclose their gender identity. The majority of participants identified as White/Caucasian (*n* = 56 [88%]). Participants’ relationship status comprised of single (*n* = 43), married/living with a partner/engaged (*n* = 12), divorced/separated (*n* = 7) and in a relationship but not cohabiting (*n* = 2). The majority were living alone (*n* = 27), 15 lived with their parents, 10 lived with their spouse/partner, 8 with friends, carers, or other relatives. Upon entry to the trial, four participants lived in either supported accommodation or on an inpatient ward. ICD-10 diagnoses included schizophrenia (*n* = 50), schizoaffective disorders (*n* = 9) persistent delusional disorders, unspecified non-organic psychosis, or transient psychotic disorders (*n* = 5).

### 2.2. Intervention

CBSPp therapy is a formulation-based approach, where initial sessions place emphasis on developing a therapeutic relationship with clients. During early sessions and throughout therapy, therapists aim to create a collaborative and trusting environment that enables clients to feel safe to engage in conversations about their suicidal experiences. Suicidal experiences, such as suicidal thoughts and acts, are explored in relation to clients’ experiences of psychosis, current problems, and life events. Cognitive-behavioral techniques, i.e., attentional control, appraisal restructuring, problem-solving skills training, behavioral techniques, and schema-focused work are then used in collaboration with the client on negative perceptions of emotion regulation, social support, and interpersonal problem solving, which, in turn, aims to alleviate appraisals of being hopeless, defeated and trapped. Towards the end of therapy schema-focused work addresses self-stigma in relation to suicidal experiences [16,21,22,40,42,43].

Participants were consistently offered up to 24, 50 min, CBSPp sessions, which were tailored to their individual needs in relation to suicide prevention. Participants attended between 5 and 24 CBSPp therapy sessions (M = 17.55, SD = 4.61), which lasted an average of 899.27 min, i.e., almost 15 h (SD = 267.38, range = 280–1380). Individual sessions lasted an average of 51.38 min (SD = 12.24, range = 5–180). Most participants met therapists in their own home throughout therapy (*n* = 36), whereas eight participants were seen at NHS community (e.g., GP clinic), outpatient or inpatient settings for all sessions. The remaining participants attended therapy conducted in a combination of settings (e.g., home and NHS settings; *n* = 20), some of whom also spoke to the therapist by telephone for a proportion, but not all, therapy sessions (*n* = 12). Data on adverse events were monitored routinely throughout the trial and there was no evidence to suggest that the therapy or therapeutic alliance was related to any serious untoward events by clients, such as, suicide attempts.

### 2.3. Therapists

Therapists who delivered CBSPp therapy were 8 individuals who met the British Association for Behavioral & Cognitive Psychotherapies standards for CBT accreditation and were also a social worker, mental health nurses, or clinical psychologists by professional training. Therapists were aged between 34 and 60 years, and seven therapists self-identified as Caucasian, three as female and four as male. Self-reported ethnicity and gender were not available for one therapist. Length of experience post-qualification was an average of 9.90 years (range = 0.5–17 years). Therapists were trained to deliver the CBSPp therapy using the treatment manual developed by the authors. Adherence to the therapy protocol was maintained through weekly group supervision, monthly individual supervision, and regular peer supervision. Group and individual supervision sessions were facilitated by two senior clinical psychologists. Therapy fidelity to the CBT approach was monitored through supervision and ratings guided by the Cognitive Therapy Scale for Psychosis [44].

### 2.4. Measures

#### 2.4.1. Working Alliance Inventory—Short Revised (WAI-SR)

Client and therapist versions of the WAI-SR [45,46] self-report questionnaire, comprising of 12 and 10 items, respectively, were used in the current study. Each item was rated on a 5-point Likert scale, where 1 (seldom) is the lowest and 5 (always) is the highest. Overall higher scores suggest that the client-therapist relationship is stronger. Items reflect three components of the alliance, namely, goals, tasks, and bond [45,46]. The client WAI-SR demonstrates excellent internal consistency (α = 0.92) and good validity (*r* = 0.80) [45]. The therapist WAI-SR also has excellent reliability (α = 0.94) good validity (*r* = 0.79) [47]. In the current study, the WAI-SR displayed excellent reliability for both client (α = 0.91) and therapist (α = 0.85) versions of the scale, respectively.

#### 2.4.2. Adult Suicide Ideation Questionnaire (ASIQ) 

The ASIQ [48] has 25 self-report items, rated on a 7-point Likert scale, where 0 (I never had this thought) is the lowest and 6 (almost every day) is the highest possible score. Overall, high scores are indicative of an individual experiencing a higher frequency and severity of suicidal thoughts. The ASIQ demonstrates excellent internal reliability in a population with mental health diagnoses, who had previously attempted suicide (α = 0.97) [48]. In the current study, the ASIQ demonstrated excellent internal reliability (α = 0.94).

#### 2.4.3. Suicide Plans and Attempts

Participants were asked to self-report ‘number of episodes when you have had a plan to take your life’ and ‘number of suicide attempts’, with both questions pertaining to the previous six months.

#### 2.4.4. Beck Hopelessness Scale (BHS) 

The BHS [49] has 20 self-report items, which are rated either true or false with a score of 1 or 0. Overall, high scores indicate greater hopelessness. The BHS had excellent internal reliability (α = 0.93) and good validity (*r* = 0.70) in a population who had experienced suicide ideation and attempts [49]. In the current study, the BHS had excellent internal reliability (α = 0.91).

#### 2.4.5. Calgary Depression Scale for Schizophrenia (CDSS) 

The CDSS [50] is observer rated and consists of 9 categories which are rated from 0 (absent) to 3 (severe). High overall scores suggest more severe depression. The CDSS demonstrates good internal reliability (α = 0.89) and discriminant validity [50]. Inter-rater reliability for the observers in the current study was excellent (Intraclass Correlation Coefficient = 0.93).

#### 2.4.6. Dose of Therapy

Due to the potential variability in session length, examining the total time spent in therapy provides a more accurate representation of contact with the therapist and total duration of therapy, compared to number of sessions (e.g., [51]). Total number of minutes of therapy sessions were recorded following each session by the therapist and this was summed for each participant.

#### 2.4.7. Missing Data

Missing ASIQ data were prorated where at least 88% of items were complete [48]. Missing data from the BHS and WAI-SRs were prorated for measures where at least 80% of the items were complete. Prorating involved calculating the mean of completed items to yield a prorated item score for missing items [52]. All participants completed the minimum percentage of items for the ASIQ, BHS and WAI-SR to be included in the analysis.

### 2.5. Procedure

Ethical approval was granted by the Greater Manchester South NRES committee (registration number 17/NW/0089).

Following eligibility screening and informed consent, participants completed baseline measures of ASIQ, BHS, CDSS and self-reported frequency of suicide plans and attempts, along with additional measures which were not used in the present study. Participants also completed suicidal experience measures upon therapy cessation. The CDSS and self-reported frequency of suicide plans and attempts were obtained through a structured interview format. The ASIQ and BHS were self-report questionnaires completed by participants. Baseline and post-therapy data were collected by research assistants, who were blind to treatment allocation. Serious adverse events, e.g., suicide attempts, were monitored and assessed for relatedness to CBSPp and/or therapeutic alliance.

During therapy, therapists and clients completed the WAI-SR around session 4 (mean weeks since starting therapy for therapist WAI-SR = 5.68 [SD = 2.79] and client WAI-SR = 6.00 (SD = 3.15)). Session 4 was selected to measure the therapeutic alliance as it is well-established that a therapeutic alliance will likely have been developed by session 4 of weekly therapy [30,33,36]. Therapists provided clients with a paper copy of the WAI-SR, to complete, with a stamped addressed envelope and reassured them that they would not see their responses.

All data were collected prior to when the UK government passed legislation for a country-wide lockdown due to the COVID-19 pandemic [41]. This was to ensure homogeneity in both therapy delivery and collection of outcome measures, which were both primarily conducted face to face.

### 2.6. Statistical Analysis

P–P plots and z scores of skewness and kurtosis were used to assess the normality of the distribution of the data [53,54]. If variables were not normally distributed and/or equal variances were not assumed between groups, bootstrapping at 1000 iterations was used [53]. A decision was made post-hoc, to correct for multiple testing and reduce the likelihood of a Type 1 error for hypotheses one and two part A, by altering the alpha level to 0.01. The alpha level for hypotheses two part B and three remains 0.05.

Pearson’s correlation coefficients were computed to address research questions one and two part A. A priori power calculation estimated that with a medium effect size of *r* = 0.33, statistical power level of 0.8 and alpha level of *p* = 0.05, a minimum of 69 participants are required. Significant bivariate correlations were selected for entry into a multiple linear regression analysis. However, to address the high frequency of nil responses, the continuous suicide attempt and suicide plan variables were transformed into dichotomous variables, i.e., no suicide attempt/plan vs. one or more suicide attempt/plan and were analyzed using an independent samples *t*-test.

A multiple hierarchical linear regression analysis was used to address research question two part B. A priori power calculation estimated that with a medium effect size of *f*^2^ = 0.15, statistical power level of 0.8, alpha level of *p* = 0.05 and 4 independent variables, a minimum of 85 participants needed to be recruited. Both multicollinearity and standardized residuals were examined to determine whether the assumptions of a multiple regression analysis were met. Acceptable multicollinearity levels included correlation coefficients below 0.8 and tolerance levels above 0.2 [53]. The hierarchical regression models were conducted with, and without, controlling for suicidal ideation, depression, and hopelessness. When controlled for, variables were entered in the following order: (1) baseline suicidal ideation; (2) depression and hopelessness; and (3) therapeutic alliance [53].

The PROCESS tool for IBM SPSS Statistics was used to conduct a moderated linear regression [55] to address research question three. A moderated linear regression was conducted, with and without, controlling for depression and hopelessness. All variables were mean centered prior to entry. Simple slopes were examined to inspect the interaction effect when number of minutes of therapy was low, mean, and high. The Johnson-Neyman method was used to define significance regions for the moderation [53].

All statistical analyses were carried out using IBM SPSS Statistics version 25 (IBM, Armonk, NY, USA). Power was calculated using G*Power 3.1 [56].

## 3. Results

Overall descriptive statistics for all variables are presented in Table 1.

### 3.1. Is There a Relationship between Suicidal Experiences Prior to Starting Therapy and the Therapeutic Alliance (Session 4)?

Baseline suicidal ideation was normally distributed. However, self-reported number of suicide attempts and suicide plans were not normally distributed. Hence, bootstrapping was used in analyses involving plans and attempts.

#### 3.1.1. Suicidal Ideation

There were no significant correlations found between suicidal ideation at baseline and the client, *r*(57) = −0.115, *p* = 0.386, 99% CI (−0.43, 0.23), or the therapist, *r*(58) = −0.034, *p* = 0.794, 95% CI (−0.36, 0.30), perceptions of the therapeutic alliance. Therefore, the severity of clients’ suicidal ideation prior to starting therapy did not appear to be related to the quality of the therapeutic alliance as perceived by either clients or therapists.

#### 3.1.2. Suicide Plans

Twenty-two participants reported no suicide plans in the 6 months prior to commencing therapy. There were no significant differences between client ratings of the therapeutic alliance regardless of whether they had not previously made suicide plans (M = 46.45, SD = 7.03, range = 37–60) or made one or more suicide plans (M = 46.82, SD = 9.02, range = 25–60), *t*(55) = −0.16, *p* = 0.863, 99% CI (−5.49, 5.52). Similarly, there were no significant differences in therapist therapeutic alliance scores of clients who had made no plans (M = 37.35, SD = 4.98, range = 26–46) and those who had made one or more suicide plans prior to starting therapy (M = 35.58, SD = 5.70, range = 23–48), *t*(56) = 1.20, *p* = 0.227, 99% CI (−2.01, 5.56).

#### 3.1.3. Suicide Attempts

**Figure 1:**
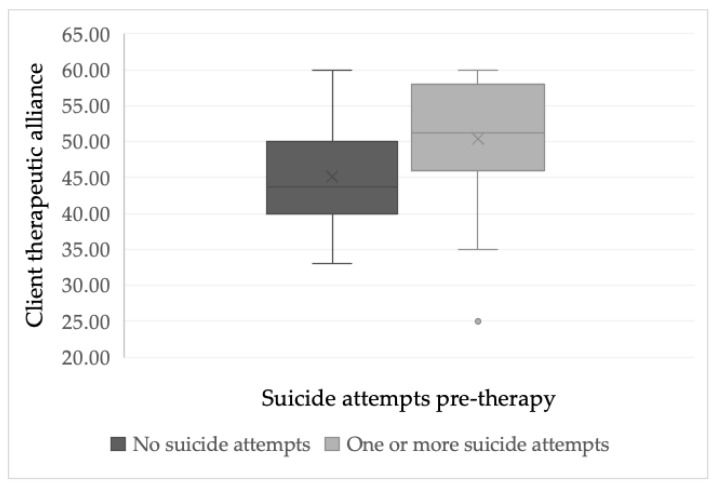
A boxplot chart illustrates the distribution of client therapeutic alliance scores between clients who had and had not attempted suicide during the 6 months prior to starting therapy.

Only 19 clients reported having recently attempted suicide, which is reflective of the rarity of suicide attempts. Using an alpha level of *p* = 0.01 to allow for multiple testing, there was no evidence found for significant differences in ratings of the therapeutic alliance measure by clients who had previously attempted suicide (M = 50.40, SD = 9.19, range = 33–60), compared to those who had not attempted suicide in the last six months (M = 45.12, SD = 6.87, range = 25–60; see Figure 1 for boxplot), *t*(56) = −2.46, *p* = 0.023, 99% CI (−10.69, 0.36). Relatedly, therapists’ therapeutic alliance scores were not significantly different when working with clients who had attempted suicide (M = 37.59, SD = 5.43, range = 30–48), compared with those who had not done so (M = 35.57, SD = 5.38, range = 23–46), *t*(57) = −1.34, *p* = 0.186, 99% CI (−5.51, 2.21).

Overall, the findings suggested that suicidal experiences in the 6 months prior to starting therapy were not related to the therapeutic alliance, as rated by both clients and therapists.

### 3.2. Does the Therapeutic Alliance Predict Suicidal Experiences Measured Post-Therapy?

#### 3.2.1. Suicidal Ideation

**Figure 2:**
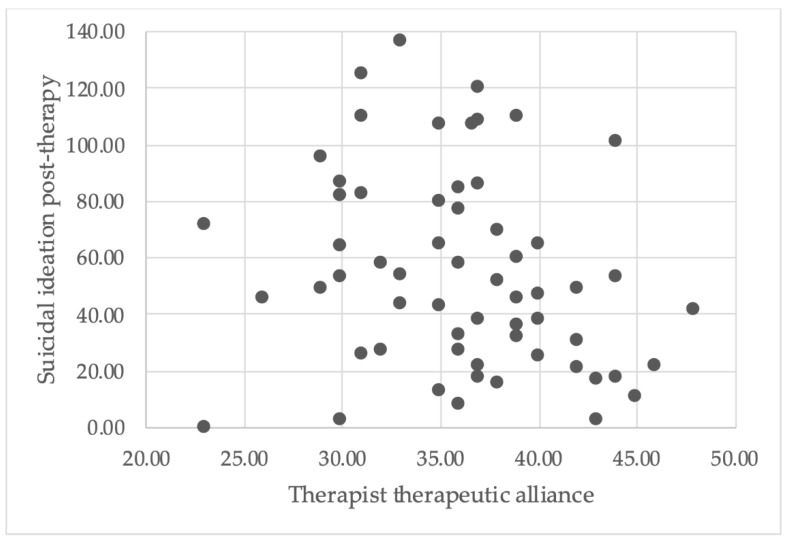
A scatterplot illustrates the relationship between therapist therapeutic alliance scores and severity of suicidal ideation post-therapy.

**Figure 3:**
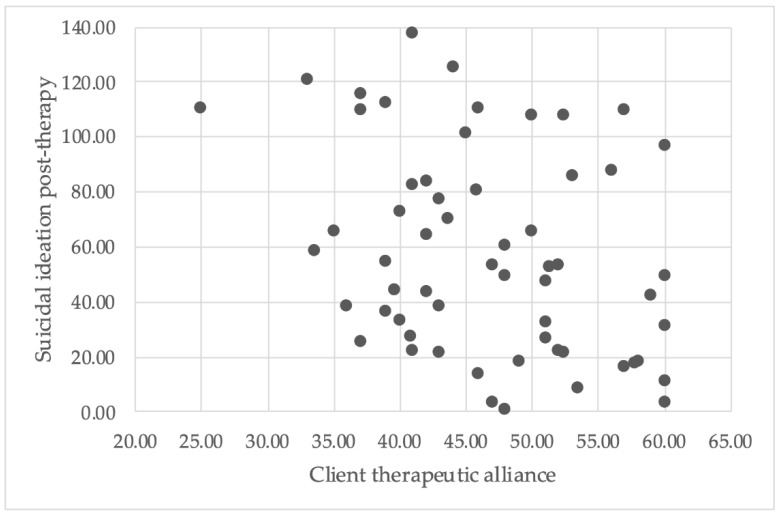
A scatterplot illustrates the relationship between client therapeutic alliance scores and severity of suicidal ideation post-therapy.

There was no significant correlation found between therapist rating of the therapeutic alliance and severity of suicidal ideation at therapy cessation (see Figure 2 for scatterplot), *r*(58) = −0.22, *p* = 0.087, 99% CI (−0.51, 0.11). Conversely, there was a significant negative correlation between the client rating of the therapeutic alliance and severity of suicidal ideation at therapy cessation (see Figure 3 for scatterplot), *r*(57) = −0.33, *p* = 0.01, 99% CI (−0.60, −0.001). In other words, a higher therapeutic alliance score was associated with less severe suicidal ideation at the end of therapy.

The association between client therapeutic alliance and suicidal ideation at the end of therapy reached significance enabling the multiple hierarchical linear regression modelling to be run. No multicollinearity nor strong inter-correlations of 0.8 or above were observed between variables. Inspection of P–P plots and z scores suggested the data were normally distributed. A Durbin–Watson value of 1.702 indicated that the data met the assumption of independent errors, and scatterplots of standardized residuals showed that the data met the assumptions of variance and linearity. Descriptive statistics and correlation coefficients for each variable in the regression model are presented in Table 2. Higher therapeutic alliance scores rated by the client significantly predicted less severe suicidal ideation upon therapy cessation (data shown in Table 3). Furthermore, in the first model, the therapeutic alliance rated by the client provided an additional 11% explanation for variation in suicidal ideation post-therapy. This reduced to 7.8% in the second model, after controlling for baseline suicidal ideation, and reduced again slightly to 6.8% in the third model, when baseline depression and hopelessness were also controlled for. Therefore, a higher therapeutic alliance score rated early in therapy by the client, predicted less severe suicidal thoughts at the end of therapy, whilst accounting for baseline suicidal ideation, depression, and hopelessness.

#### 3.2.2. Suicide Plans

It should be noted that only 33 of the 64 participants had reported making suicide plans during the delivery of the therapy. There were no significant differences in client ratings of the initial therapeutic alliance between those who made no suicide plans (M = 47.35, SD = 7.94, range = 35–60) and clients who had made one or more plans (M = 45.70, SD = 8.80, range = 25–60) during the 6 months when therapy had taken place, *t*(54) = 0.74, *p* = 0.454, 99% CI (−3.69, 8.01). Similarly, there were no significant differences in therapist ratings of the early therapeutic alliance between clients with (M = 35.42, SD = 4.41, range = 29–44) and without suicide plans (M = 37.31, SD = 5.70, range = 23–48), *t*(55) = 1.38, *p* = 0.184, 99% CI (−2.01, 5.14).

#### 3.2.3. Suicide Attempts

There were no significant differences between clients who had not attempted suicide (M = 46.17, SD = 8.27, range = 25–60) and those who had made one or more suicide attempts (M = 48.33, SD = 8.44, range = 33–60), during therapy, in their early therapeutic alliance scores, *t*(55) = −0.72, *p* = 0.463, 99% CI (−9.62, 6.64). Likewise, there were no significant differences in therapist ratings of the therapeutic alliance with clients who had made no suicide attempts (M = 36.51, SD = 5.24, range = 23–48) and those who had made one or more suicide attempts (M = 35.25, SD = 5.28, range = 29–44) in the 6 months prior to therapy ending, *t*(56) = 0.63, *p* = 0.529, 99% CI (−4.68, 6.36).

In summary, a higher therapeutic alliance score, rated by the client, predicted lower severity of suicidal ideation at the end of therapy. However, the client and therapist ratings of the therapeutic alliance did not appear to relate to suicide attempts and plans at the end of therapy.

### 3.3. Does the Number of Minutes of CBSPp Therapy Session Attendance Amplify the Relationship between the Therapeutic Alliance and Suicidal Experiences Post-Therapy?

**Figure 4:**
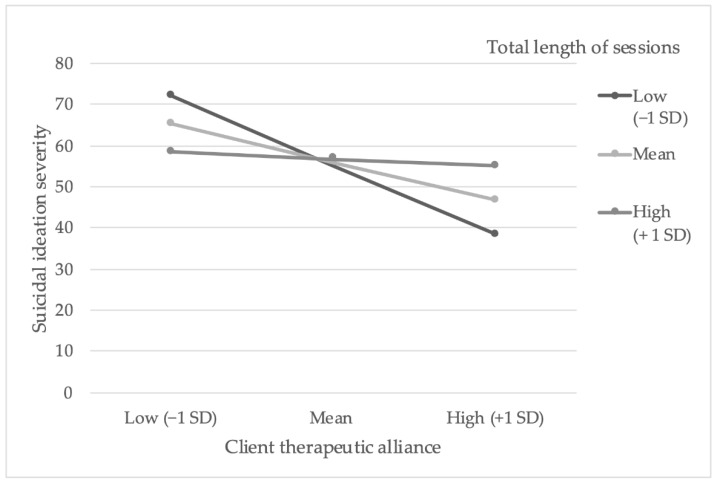
Simple slopes equations of the regression of the therapeutic alliance on suicidal ideation at high, mean, and low levels of total number of minutes of therapy. Note. High, mean, and low values of total number of minutes of therapy are defined as plus and minus 1 SD of the mean (−269.18, 0, 269.18). Low, mean, and high values of the therapeutic alliance score are defined as plus and minus 1 SD of the mean (−8.14, 0, 8.14).

There was a significant main effect of client therapeutic alliance on severity of suicidal ideation (data presented in Table 4), and there was also a trend towards a significant interaction effect of the moderator, i.e., total number of minutes of therapy. To investigate the potential interaction effect further, simple slopes analyses were examined for three models (see Figure 4). The results indicated that when total number of minutes of psychotherapy were short or at the mean value, there was a significant negative relationship between client therapeutic alliance score and severity of suicidal ideation upon therapy cessation. However, when total number of minutes of therapy were long, there was not a significant relationship between client rating of the therapeutic alliance and severity of suicidal ideation upon therapy cessation. Therefore, the moderation model suggested that a higher score on the therapeutic alliance measure, rated by the client, predicted less severe suicidal ideation, but only when total number of minutes of therapy were short or average.

On further examination of these data, and with application of the Johnson–Neyman method to define significance regions, the final point where total number of minutes of therapy significantly influenced the relationship between therapeutic alliance and suicidal ideation was 24.41 min above the mean total length of therapy sessions (i.e., 923.68 min). Therefore, as total number of minutes of therapy increased above 924 min (i.e., 15.4 h), the therapeutic alliance score, as rated by the client, no longer predicted lower severity of suicidal ideation.

Moreover, when further covariates, specifically, baseline depression and hopelessness, were added to the model, there was no longer a trend towards significance for the interaction effect of total number of minutes of therapy.

## 4. Discussion

The current study addressed a major gap in the literature by examining the relationship between the therapeutic alliance in therapy and suicidal experiences (pre- and post-therapy) in a population with non-affective psychosis. Further, it provides one key novel insight into the influence that therapy dosage has upon this relationship.

It was evident that suicidal ideation, plans, and attempts occurring prior to therapy did not have a positive or negative relationship with client or therapist perceptions of the subsequent therapeutic alliance. Such finding is reflective of the wider suicide-specific literature, when the client rated alliance was measured at session 3, 4, or one month into therapy [31,32,57]. CBSPp therapy may have helped to address previously unmet needs and provided an opportunity to discuss recent painful events and circumstances surrounding suicidal experiences [16,25,42]. Clients may not have wished to share such experiences with their family or friends for fear of being seen as a burden and/or due to the shame and stigma associated with talking about suicidal experiences [25,26,27]. We acknowledge that the analysis was underpowered, which offers another reason as to why a significant relationship was not detected. Nevertheless, future research should adopt qualitative methods to explore how the therapeutic alliance develops in CBSPp as this may help to further explain these findings.

In line with the current literature, a stronger, client perceived, therapeutic alliance developed early on in CBSPp predicted less severe suicidal ideation, even after controlling for the influence of pre-therapy suicidal ideation, depression, and hopelessness. This finding is important because suicidal ideation, depression and hopelessness are established predictors of suicidal experiences, including those with non-affective psychosis [3,5,6,7,10,11,12,13]. Furthermore, suicidal ideation is associated with immense distress and may be a precursor of suicide plans and attempts [15,16]. Therefore, if a more robust therapeutic alliance is developed and suicidal ideation less severe, this may in turn, reduce the likelihood of suicide plans and attempts. Additionally, the current study has shown that this finding can be applied to a population with non-affective psychosis, which was a notable gap in the literature [39].

One key novel insight was gained into the role of dose of therapy in the relationship between the therapeutic alliance and suicidal ideation. There was a trend towards significance in the overall interaction effect. Nonetheless, the dose of therapy appeared to have amplified the predictive relationship of a stronger therapeutic alliance and lower severity in suicidal thoughts, for clients spending less than 15.4 h in CBSPp therapy. This somewhat contradicts previous CBTp literature [36] which does not suggest a ceiling effect of dose of therapy on the relationship between a strong therapeutic alliance and better outcome. However, the present study provides a preliminary suggestion for how much time clients should optimally spend in CBSPp therapy in order to maximally benefit, a finding of huge importance for service planning. Consequently, the findings also place further emphasis on the importance of developing a stronger therapeutic alliance, from the client’s point of view, and monitoring the strength of an alliance alongside total number of minutes spent in therapy, in order to prevent the possibility of adverse effects or potential harm occurring during therapy.

The aforementioned findings should be considered in light of five key limitations of the current study. First, it should be noted that the analyses in the present study were likely underpowered and should be replicated in larger samples. Nevertheless, strong findings were produced for the alliance-outcome relationship, which were concurrent with the suicide-specific alliance-outcome literature (e.g., [31,39]). The current study findings were also consistent with the wider alliance-outcome literature, which suggests that such relationship is both transdiagnostic and applicable across psychological therapy models [33]. In addition, the moderated regression findings provide a novel contribution to the literature.

Second, the design and statistical analyses were unable to provide evidence for causality or the direction of causality. Up to two time points were used to measure data in the current study, which may not fully capture possible fluctuations in the therapeutic alliance and suicidal experiences and how they relate to each other session-by-session. Future studies should adopt session-by-session ratings of therapeutic alliance and suicidal experiences during both CBSPp and a control therapy (e.g., supportive counselling) and use hierarchical linear modelling, path analysis or time series analysis. This approach will better capture the nuances of the relationship between the therapeutic alliance and suicidal experiences throughout therapy and provide some evidence for causality and the direction of causality.

Third, eight therapists delivered CBSPp therapy, which could have introduced variability in alliance development and client suicidal experience outcomes. That said, therapists received a robust training package in the delivery of CBSPp by two senior clinical psychologists and regular group, individual and peer supervision to ensure fidelity. Challenges in developing a robust therapeutic alliance were discussed in group supervision.

Fourth, there was a lack of diversity in both therapist and client samples thus limiting the applicability of the current findings to ethnic minority communities, for example. Additionally, the study was conducted in the UK and would not be appropriate to generalize the findings to other countries. This is due to cultural differences in beliefs about factors which contribute to severe mental health problems (e.g., psychosis), and the perception, offer, accessibility, and delivery of psychological therapy [58,59,60,61]. Therefore, future studies should ensure that therapists and service user participants from minority groups living in the UK are pro-actively recruited and therapy is appropriately adapted to suit their cultural needs [59,60,61,62]. Relatedly, it is important to consider how demographic factors such as ethnicity, age, and gender of therapists and clients contribute to the development and maintenance of the therapeutic alliance [63,64]. This is currently an under researched area in people with suicidal experiences [39].

Fifth, there were some missing client and therapist WAI data, which could have introduced bias to the findings. Additionally, five participants received fewer than 10 therapy sessions and over half of clients met with the therapist in their own home across all sessions. Clients who perceive the therapeutic alliance as poorer are more likely to discontinue therapy [65,66] or may not be willing to complete the therapeutic alliance or outcome assessment measures [67]. Consequently, the current findings may be over-representative of clients who were more willing to engage with therapy, may have felt more comfortable in the therapy setting and had a stronger alliance with the therapist. Nevertheless, there was a consistent therapy offer provided to participants, with variable uptake, which is reflective of UK-based mental health services.

## 5. Conclusions

A stronger, client viewed, therapeutic alliance was predictive of lower severity in suicidal ideation, which appeared to be moderated by total number of minutes spent in therapy. The current study has demonstrated that this finding can be applied to a population with non-affective psychosis, i.e., those who have severe mental health problems, addressing an important gap in the literature. It is indicated that therapists working with suicidal clients could place an emphasis on building and maintaining a stronger therapeutic alliance and understand what that means from the client’s point of view. The present study also provides encouraging results and suggests that future, larger-scale, studies should adopt a longitudinal design, which would improve the evidence-base, better capture the nuances of the relationship between the therapeutic alliance and suicidal experiences throughout therapy and provide some evidence for causality and the direction of causality.

## Figures and Tables

**Table 1:** Full sample descriptive statistics for client and therapist therapeutic alliance (session 4), suicidal ideation, suicide plans, suicide attempts (pre- and post-therapy), and depression and hopelessness (pre-therapy).

	*N*	M (SD)	Range
1. Client therapeutic alliance	59	46.62 (8.14)	25–60
2. Therapist therapeutic alliance	60	36.14 (5.41)	23–48
3. Suicidal ideation (pre-therapy)	64	78.44 (31.86)	9–129
4. Suicidal ideation (post-therapy)	64	56.78 (35.87)	0–137
5. Depression (pre-therapy)	64	12.47 (4.62)	3–21
6. Hopelessness (pre-therapy)	64	13.19 (5.55)	1–20
7. Suicide plans (pre-therapy)	62	19.35 (42.70)	0–180
No suicide plans	24	0	0
One or more suicide plans	38	38.58 (51.08) ^a^	1–180
8. Suicide plans (post-therapy)	61	10.59 (34.01)	0–180
No suicide plans	33	0	0
One or more suicide plans	28	23.07 (47.66) ^a^	1–180
9. Suicide attempts (pre-therapy)	63	1.41 (6.33)	0–50
No suicide attempts	43	0	0
One or more suicide attempts	20	4.45 (10.79) ^a^	1–50
10. Suicide attempts (post-therapy)	62	0.68 (2.89)	0–21
No suicide attempts	53	0	0
One or more suicide attempts	9	4.67 (6.54) ^a^	1–21

Note. ^a^ Mode is 1.

**Table 2:** Descriptive statistics and correlation coefficients for variables in hierarchical linear regression model 2.

Continuous Variables	*N*	M (SD)	Range	2	3	4	5
1. Client therapeutic alliance	59	46.62 (8.14)	25–60	−0.12	−0.33 *	−0.17	−0.18
2. Suicidal ideation (pre-therapy)	59	77.03 (32.07)	9–129	-	0.48 *	0.41 *	0.61 *
3. Suicidal ideation (post-therapy)	59	57.14 (36.92)	0–137		-	0.23	0.44 *
4. Depression (pre-therapy)	59	12.29 (4.54)	3–21			-	0.53 *
5. Hopelessness (pre-therapy)	59	12.80 (5.56)	1–20				-

Note. * *p* < 0.01.

**Table 3:** Results of the hierarchical linear regression model examining the predictive relationship between the early therapeutic alliance and suicidal ideation post-therapy after controlling for suicidal ideation pre-therapy, depression, and hopelessness.

Model		B (95% CI)	SE B	β	*p*
Model 1 ^a^: outcome of post-therapy suicidal ideation	Step 1				
	Constant	127.42 (73.73, 181.11)	26.81		0.000
	Client WAI-SR	−1.51 (−2.64, −0.37)	0.57	−0.33	0.010
Model 2 ^b^: outcome of post-therapy suicidal ideation, whilst controlling for baseline suicidal ideation	Step 1				
	Constant	14.53 (−7.79, 36.85)	11.15		0.198
	ASIQ (pre-therapy)	0.55 (0.29, 0.82)	0.13	0.48	0.001
	Step 2				
	Constant	76.80 (22.73, 130.86)	26.99		0.006
	ASIQ (pre-therapy)	0.52 (0.29, 0.77)	0.13	0.45	0.001
	Client WAI-SR	−1.27 (−2.29, −0.26)	0.51	−0.28	0.015
Model 3 ^c^: outcome of post-therapy suicidal ideation, whilst controlling for baseline suicidal ideation, depression, and hopelessness	Step 1				
	Constant	14.53 (−7.79, 36.85)	11.15		0.198
	ASIQ (pre-therapy)	0.55 (0.29, 0.82)	0.13	0.48	0.001
	Step 2				
	Constant	9.31 (−18.30, 36.92)	13.78		0.502
	ASIQ (pre-therapy)	0.40 (0.06, 0.73)	0.17	0.34	0.023
	CDSS (pre-therapy)	−0.35 (−2.59, 1.89)	1.12	−0.04	0.753
	BHS (pre-therapy)	1.70 (−0.40, 3.79)	1.05	0.26	0.110
	Step 3				
	Constant	70.98 (11.96, 130.00)	29.44		0.019
	ASIQ (pre-therapy)	0.40 (0.07, 0.72)	0.16	0.34	0.018
	CDSS (pre-therapy)	−0.58 (−2.74, 1.58)	1.08	−0.07	0.591
	BHS (pre-therapy)	1.49 (−0.54, 3.51)	1.01	0.22	0.148
	Client WAI-SR	−1.21 (−2.23, −0.18)	0.51	−0.27	0.023

Note. ^a^ Model 1: R^2^ = 0.110, *p* = 0.010 for step 1; ^b^ Model 2: R^2^ = 0.231, *p* = 0.001 for step 1; ΔR^2^ = 0.078, *p* = 0.015 for step 2; ^c^ Model 3: R^2^ = 0.231, *p* = 0.001 for step 1; ΔR^2^ = 0.037, *p* = 0.261 for step 2; ΔR^2^ = 0.068, *p* = 0.023 for step 3. WAI-SR = Working Alliance Inventory—Short Revised; ASIQ = Adult Suicide Ideation Questionnaire; BHS = Beck Hopelessness Scale; CDSS = Calgary Depression Scale for Schizophrenia.

**Table 4:** Results of the moderated linear regression model examining the effect of total length of therapy sessions on the relationship between the client therapeutic alliance and suicidal ideation post-therapy.

Model		b (95% CI)	SE B	t	*p*
Model 1 ^a^: baseline suicidal ideation controlled for	Constant	16.80 (−5.33, 38.93)	11.04	1.52	0.198
	Client WAI-SR (centered)	−1.14 (−2.18, −0.10)	0.52	−2.20	0.032
	Total number of minutes of therapy (centered)	0.003 (−0.03, 0.03)	0.02	0.12	0.861
	Client WAI-SR x Total number of minutes of therapy	0.003 (−0.0003, 0.007)	0.01	1.85	0.07
	ASIQ (pre-therapy)	0.51 (0.24, 0.78)	0.13	3.81	0.001
Model 2 ^b^: baseline suicidal ideation, depression and hopelessness controlled for	Constant	19.41 (−10.92, 47.74)	14.62	1.26	0.214
	Client WAI-SR (centered)	−1.13 (−2.19, −0.07)	0.53	−2.13	0.038
	Total number of minutes of therapy (centered)	0.002 (−0.03, 0.04)	0.02	0.13	0.896
	Client therapeutic alliance x Total number of minutes of therapy	0.003 (−0.0003, 0.007)	0.01	1.57	0.123
	ASIQ (pre-therapy)	0.45 (0.12, 0.78)	0.17	2.71	0.009
	CDSS (pre-therapy)	−0.85 (−3.06, 1.37)	1.10	−0.77	0.446
	BHS (pre-therapy)	1.05 (−1.06, 3.16)	1.05	1.00	0.322

Note. ^a^ Model 1: R^2^ = 0.35, p = 0.001; ΔR^2^ = 0.041, *p* = 0.07 for interaction; ^b^ Model 2: R^2^ = 0.37, *p* = 0.001; ΔR^2^ = 0.03, *p* = 0.123 for interaction. WAI-SR = Working Alliance Inventory—Short Revised; ASIQ = Adult Suicide Ideation Questionnaire; BHS = Beck Hopelessness Scale; CDSS = Calgary Depression Scale for Schizophrenia.

## Data Availability

The data are not publicly available due to sensitive information.
